# Vibration Excitation and Suppression of a Composite Laminate Plate Using Piezoelectric Actuators

**DOI:** 10.3390/ma15062027

**Published:** 2022-03-09

**Authors:** Shiuh-Chuan Her, Han-Yung Chen

**Affiliations:** Department of Mechanical Engineering, Yuan-Ze University, 135 Yuan-Tong Road, Taoyuan 32003, Taiwan; s965025@mail.yzu.edu.tw

**Keywords:** piezoelectric actuator, composite laminate plate, vibration, suppression

## Abstract

Piezoelectric (PZT) actuators bonded on a structure can be used to generate deformation and excite vibration for the shape control and vibration suppression, respectively. This article proposes a theoretical model for predicting vibrational response of a composite laminate plate with PZT actuators. The bending moment induced by the PZT actuator was obtained and applied on the composite laminate plate. Utilizing composite mechanics and plate theory, an analytical solution of the vibrational response of a composite laminate plate excited by the PZT actuator with oscillating voltage was derived. Furthermore, the finite element analysis using ANSYS software (2019 version) was carried out to compare with the proposed model with a good agreement. A parametric study was performed to investigate the influences of PZT location and frequency on the vibration. Numerical results illustrate that mode can be selectively excited provided the PZT actuator is placed in an appropriate location. Moreover, the proposed model was employed to predict the effectiveness of vibration suppression by distributed PZT actuators. The novelty of this work is that a complicated coupling problem between the composite plate and bonded PZT actuator is resolved into two simple problems, leading to a simple analytical solution for the vibrational response of a composite plate induced by PZT actuators. The proposed model has been successfully demonstrated its applications on the vibration excitation and suppression of a composite laminate plate.

## 1. Introduction

The use of composite material in industries such as aerospace, sports, and automobile has been increasing rapidly over the last decade due to its high strength-to-weight ratio. One of the main advantages of the composite over conventional materials is that it can be tailored to meet the certain requirements of a specific application, for example, minimum weight, maximum failure load, and maximum fundamental frequency [1,2,3]. The incorporation of actuators and sensors to perform structural health monitoring has introduced a new class of structures known as smart structures. A smart structure has the capability of providing appropriate response to the either external or internal environmental changes through the incorporated sensors and actuators. One of the most promising smart structures is a composite laminate equipped with distributed sensors and actuators. A variety of materials have been proposed for sensors and actuators, such as piezoelectric ceramics [4], magnetostrictive [5], shape memory alloy [6], and electrostrictive [7]. PZT has the advantages of light weight, fast response, low cost and easy implementation, which can be utilized for shape and vibration control. Moreover, PZTs can be attached on or embedded into a host structure with minimum modification of the original structure [8].

The use of composite laminates is very appropriate to integrate with PZT actuators to form a smart structure. The sensing and actuating functions of PZT are inherent to the composite material, which provide the desired capability for structural control applications [9]. Composite structures incorporated with PZT actuators have received a great amount of attention in structural engineering with applications such as shape control [10], noise reduction [11], and vibration suppression [12]. A PZT actuator is capable of providing active control for structures. For the vibration control, the external disturbance is suppressed to maintain the structure in its equilibrium position, while shape control enables the structure to deform in a desired and proper shape [13]. Schulz et al. [14] studied the influence of the allocation of piezoelectric patches in composite structures for vibration control using a finite element method and a linear quadratic regulator. Kerboua et al. [15] reported a 42% reduction in bending vibration for a cantilever beam by a PZT patch. The equation of motion of a composite beam bonded with a PZT patch was derived using Hamilton’s principle and Galerkin’s method. Bao et al. [16] developed a smart beam structure equipped with periodic piezoelectric arrays for wave propagation and structural vibration suppression. Song et al. [17] utilized PZT actuators to control the shape of an antenna reflector. A finite element model of the integrated reflector–actuator system was developed using piezoelectric constitutive equations and the principle of virtual work. Wang et al. [18] proposed a general genetic algorithm to maximize static shape control of a laminated composite structure using piezocomposite actuators. Wang et al. [19] investigated the optimal configuration of piezocomposite actuators for bending and twisting vibration control of plate structures. Bruant and Proslier [20] employed piezoelectric actuators for active vibration control of thin, axial, functionally graded beams. Kuriakose and Sreehari [21] experimentally demonstrated that piezoelectric patches bonded on an airplane wing panel can actually sense the amount of vibration and suppress it using an active vibration control system. Ding [22] employed the principle of superposition to solve the coupled vibrational problems in lateral and thickness directions of piezoelectric array elements. Yu and Zhou [23] investigated the power generation mechanism of a composite structure excited by piezoelectric devices for vibratory energy harvesting. Yayli [24] studied the axial vibration of a Rayleigh nanorod using a general eigenvalue algorithm. Zappino and Carrera [25] developed a method for numerical modeling of piezoelectric transducers based on Carrera’s Unified Formulation for the health monitoring of layered structures. Alagoz and Apak [26] investigated the removal of dust particles from solar panel surfaces by applying surface acoustic waves with piezoelectric actuators. Xu et al. [27] developed a nonresonant piezoelectric motor using horizontal and vertical PZT elements to obtain the desired oblique linear motion with a long stroke and a precise driving ability. Garg and Dwivedy [28] designed a piezoelectric energy harvester comprising a cantilever beam equipped with a PZT patch to convert vibrational energy into electric energy. Madeira and Araujo [29] investigated optimal distribution of surface-bonded PZT actuators for noise attenuation in laminated sandwich panels.

In this work, the vibrational response of a composite laminate plate excited by PZT actuators is investigated. The primary objective of this study is to actively control the vibration of a composite laminate plate through the attached PZT actuators. Theoretical prediction of the vibrational response of a composite laminate plate was developed based on the composite mechanics and piezoelectric theory under electric loading. The influences of the frequency and location of the PZT actuators on the vibrational responses were investigated. Numerical examples were presented to demonstrate the feasibility of active vibration control by exciting or suppressing the desired vibration mode. The finite element method is a widely accepted and useful tool for analyzing complicated structures. Numerous studies have been completed analyzing piezoelectric structures using the finite element method [30,31,32]. Among commercially available finite element analysis (FEA) codes, ANSYS is well-known, and its ability to model piezoelectric materials has been demonstrated [4]. The theoretical prediction was compared with the finite element method using ANSYS software.

## 2. Bending Moments Exerted on Composite Laminate by PZT Actuators

In this study, PZT actuators were attached on a cross-ply composite laminate plate and activated by an electric loading. The bending moments applied on the composite laminate induced by the PZT actuators have been derived by Her and Chen [33] as follows:

(1) $$m_{x}=\int_{z_{a}}^{z_{b}}E_{x}^{Pe}{\left(\frac{z-t_{bx}}{r_{x}}\right)}^{2}dz$$

(2) $$m_{y}=\int_{z_{a}}^{z_{b}}E_{y}^{Pe}{\left(\frac{z-t_{by}}{r_{y}}\right)}^{2}dz$$

where $E_{x}^{pe}{\;\mathrm{and}\;E}_{y}^{pe}$ represent the elastic moduli of the piezoelectric material along the *x* and *y* directions, respectively; $r_{x}$ and $r_{y}$ are the radii of curvatures of the composite laminate plate in the *x*–*z* and *y*–*z* planes, respectively; $t_{bx}$ and $t_{by}$ are the positions of neutral axes in the *x*–*z* and *y*–*z* planes, respectively; and *z* denotes the coordinate in the thickness direction. $z_{b}{\;\mathrm{and}\;z}_{a}$ are the positions of the top and bottom surfaces of the PZT actuator in the thickness direction, respectively.

**Figure 1:**
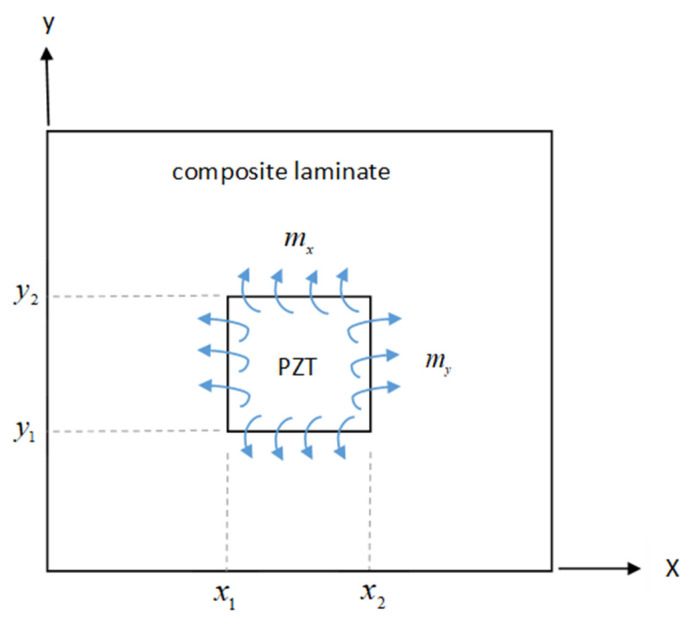
Schematic diagram of bending moments acting on the composite laminate.

A schematic diagram of bending moments acting on the composite laminate induced by the PZT actuator are illustrated in Figure 1.

## 3. Vibrational Analysis of a Composite Laminate Plate

The PZT actuator activated by an electric voltage will induce bending moments on the composite laminate as illustrated in Figure 1 and expressed in Equations (1) and (2). If the input electric voltage is a sinusoidal function sin(ωt) with an angular frequency of ω, the bending moments will vibrate with the same frequency as the electric voltage, resulting in a simply harmonic vibration of the composite laminate. Based on the composite laminate plate theory, the equation of motion of the cross-ply composite laminate can be expressed in terms of the flexural displacement and external bending moments ($m_{x}\;\mathrm{and}\;m_{y}$) induced by the PZT actuator as follows [34]:

(3) $$D_{11}\frac{\partial^{4}w}{\partial x^{4}}+2H_{1}\frac{\partial^{4}w}{\partial x^{2}\partial y^{2}}+D_{22}\frac{\partial^{4}w}{\partial y^{4}}+m^{\prime \prime }\ddot{w}=P\left(x,y,t\right)$$

(4) $$P\left(x,y,t\right)=\frac{\partial^{2}m_{x}}{\partial x^{2}}+\frac{\partial^{2}m_{y}}{\partial y^{2}}$$

(5) $$H_{1}=D_{12}+2D_{66}$$

(6) $$\left[\begin{array}{ccc} D_{11} & D_{12} & D_{16}\\ D_{12} & D_{22} & D_{26}\\ D_{15} & D_{26} & D_{66} \end{array}\right]=\frac{1}{3}\overset{N}{\underset{i=1}{\sum }}\left[\begin{array}{ccc} Q_{11} & Q_{12} & Q_{16}\\ Q_{12} & Q_{22} & Q_{26}\\ Q_{16} & Q_{26} & Q_{66} \end{array}\right]\left(z_{i}^{3}-z_{i-1}^{3}\right)$$

where $m^{\prime \prime }\;\mathrm{and}\;w$ represent the area mass density and flexural displacement of the composite laminate plate, respectively; *i* and *N* are the *i*-th ply and total number of plies in the composite laminate, respectively; ${\left[Q\right]}_{3\times 3}$ denotes the stiffness matrix of the *i*-th ply; $z_{i}\;\mathrm{and}\;z_{i-1}$ are positions of the top and bottom surfaces of the *i*-th ply in the thickness direction, respectively; and $Q_{16}=Q_{26}=0$ for the cross-ply composite laminate.

Bending moments acting on the composite laminate plate by the PZT actuator as depicted in Figure 1 are incorporated with unit step functions. Unit step functions were introduced to specify the locations where the bending moments were exerted on the composite laminate plate by the PZT actuator. The loading *P*(*x*,*y*,*t*) shown in Equation (3) is rewritten as

(7) $$p\left(x,y,t\right)=\frac{\partial^{2}m_{x}}{\partial x^{2}}+\frac{\partial^{2}m_{y}}{\partial y^{2}}=\frac{\partial^{2}}{\partial x^{2}}\{m_{x}\left[h\left(x-x_{1}\right)-h\left(x-x_{2}\right)\right]\left[h\left(y-y_{1}\right)-\;h\left(y-y_{2}\right)\right]\}+\frac{\partial^{2}}{\partial y^{2}}\left\{m_{y}\left[h\left(x-x_{1}\right)-h\left(x-x_{2}\right)\right]\left[h\left(y-y_{1}\right)-h\left(y-y_{2}\right)\right]\right\}\;=m_{x}\left[\delta^{\prime }\left(x-x_{1}\right)-\delta^{\prime }\left(x-x_{2}\right)\right]\left[h\left(y-y_{1}\right)-h\left(y-y_{2}\right)\right]\;+m_{y}\left[h\left(x-x_{1}\right)-h\left(x-x_{2}\right)\right]\left[\delta^{\prime }\left(y-y_{1}\right)-\delta^{\prime }\left(y-y_{2}\right)\right]$$

As the PZT actuator is activated by an oscillating voltage with a sinusoidal function sin(ωt), the bending moments will vibrate with the same frequency as the input voltage. The loading function *P*(*x*,*y*,*t*) in Equation (7) is as follows:

(8) P(x,y,t)=P(x,y)sinωt

(9) $$P\left(x,y\right)=m_{x}\left[\delta^{\prime }\left(x-x_{1}\right)-\delta^{\prime }\left(x-x_{2}\right)\right]\left[h\left(y-y_{1}\right)-h\left(y-y_{2}\right)\right]\;\;\;\;\;\;\;\;\;\;\;\;\;\;\;\;\;\;\;\;\;+m_{y}\left[h\left(x-x_{1}\right)-h\left(x-x_{2}\right)\right]\left[\delta^{\prime }\left(y-y_{1}\right)-\delta^{\prime }\left(y-y_{2}\right)\right]$$

For a simply supported plate, the flexural displacement can be written in terms of the Fourier sine series. Since it can satisfy the simply supported boundary conditions, both the displacement and moment are equal to zero along the boundaries.

(10) $$𝓌\left(x,y,t\right)=\left[\overset{\infty }{\underset{m=1}{\sum }}\overset{\infty }{\underset{n=1}{\sum }}W_{mn}\sin\frac{m\pi x}{a}\sin\frac{n\pi y}{b}\right]\sin\left(\omega t\right)$$

where *a* and *b* denote the length and width of the plate, respectively, and $W_{mn}$ is the constant associated with the Fourier sine series.

The acceleration can be obtained by differentiating flexural displacement as shown in Equation (10) with respect to time twice, yielding

(11) $$a\left(x,y,t\right)=\left[-\overset{\infty }{\underset{m=1}{\sum }}\overset{\infty }{\underset{n=1}{\sum }}W_{mn}\sin\frac{m\pi x}{a}\sin\frac{n\pi y}{b}\right]\omega^{2}\sin\left(\omega t\right)$$

The loading function *P*(*x*,*y*,*t*) presented in Equation (8) can also be written in terms of the Fourier sine series as follows:

(12) $$P\left(x,y,t\right)=\left[\overset{\infty }{\underset{m=1}{\sum }}\overset{\infty }{\underset{n=1}{\sum }}P_{mn}\sin\frac{m\pi x}{a}\sin\frac{n\pi y}{b}\right]\sin\omega t$$

(13) $$P_{mn}=\int_{0}^{b}\int_{0}^{a}P\left(x,y\right)\sin\frac{m\pi x}{a}\sin\frac{n\pi y}{b}dxdy\;=\frac{4}{a\times b}\left[-\frac{m_{y}\gamma_{m}{}^{2}+m_{x}\gamma_{n}{}^{2}}{\gamma_{m}\gamma_{n}}\left(\cos\gamma_{m}x_{1}-\cos\gamma_{m}x_{2}\right)\left(\cos\gamma_{n}y_{1}-\cos\gamma_{n}y_{2}\right)\right]\;\gamma_{m}=\frac{m\pi }{a},\;\gamma_{n}=\frac{n\pi }{b}$$

Substituting the flexural displacement Equation (10) and loading Equation (12) into Equation (3) leads to

(14) $$\left[D_{11}\frac{m^{4}\pi^{4}}{a^{4}}+2H_{1}\frac{m^{2}\pi^{2}}{a^{2}}\frac{n^{2}\pi^{2}}{b^{2}}+D_{22}\frac{n^{4}\pi^{4}}{b^{4}}-m^{\prime \prime }\omega^{2}\right]W_{mn}=P_{mn}$$

The constant $W_{mn}$ can then be determined.

(15) $$W_{mn}=\frac{P_{mn}}{m^{\prime \prime }\left(\omega_{mn}^{2}-\omega^{2}\right)}$$

(16) $$\omega_{mn}=\pi^{2}\sqrt{\frac{D_{11}\frac{m^{4}}{a^{4}}+2H_{1}\frac{m^{2}n^{2}}{a^{2}b^{2}}+D_{22}\frac{n^{4}}{b^{4}}}{m^{\prime \prime }}}$$

Thus, the vibrational response of the composite laminate plate excited by the PZT actuator can be obtained by substituting the constant $W_{mn}$ Equation (15) into Equation (10), while the natural frequency can be determined using Equation (16).

## 4. Numerical Results and Verification

**Figure 2:**
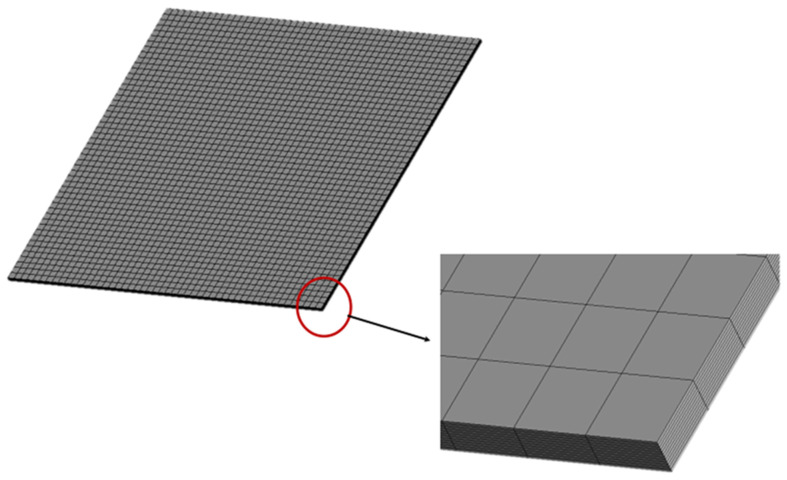
Finite element mesh.

**Figure 3:**
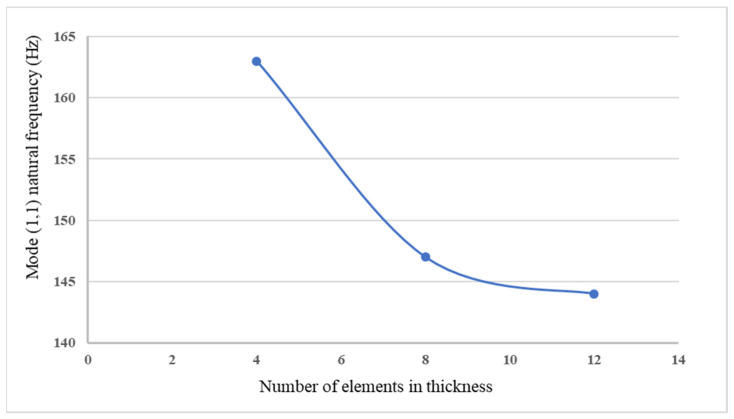
Mode (1,1) natural frequency of the composite laminate varying with the number of elements in the thickness.

Numerical examples are presented to demonstrate the capability for the excitation of a cross-ply composite laminate plate by a PZT actuator. Several parameters such as PZT size, location, frequency, layup, and stacking sequence can affect the PZT actuation. In this work, the influences of PZT size, location, and voltage frequency on the vibrational response of a composite laminate plate are investigated. The composite material adopted in this study was Boron/Al. The material properties of Boron/Al are as follows [33]: longitudinal modulus *E*_1_ = 227 GPa, transverse modulus *E*_2_ = 139 GPa, shear modulus *G*_12_ = 57.6 GPa, shear modulus *G*_23_ = 49.1 GPa, Poisson’s ratio *v*_12_ = 0.24, Poisson’s ratio *v*_23_ = 0.36, and density $\rho =2.65\;g/{\mathrm{cm}}^{3}$. The length, width, and thickness of the rectangular composite laminate plate are 500 mm, 500 mm, and 5 mm, respectively. The material properties [33] for PZT are: density $\rho =7.28\;g/{\mathrm{cm}}^{3}$, Young’s modulus *E_pe_* = 78 GPa, Poisson’s ratio *v_pe_* *=* 0.31, piezoelectric constant *d_31_* = −1.22 × 10^−10^ V/m, and thickness *t_pe_* = 0.5 mm. The finite element method was employed to compare with the proposed approach. The commercial finite element software ANSYS was used. ANSYS is a general-purpose finite element modeling package for numerically solving a wide variety of structural problems including static/dynamic, stress analysis, heat transfer, and fluid problems, as well as piezoelectric, acoustic, and electromagnetic problems. In the finite element analysis, the SOLID45 element with eight 3D nodes and orthotropic material properties was selected for the composite laminate plate. SOLID5 element was used for the piezoelectric material. It is a 3D element with eight nodes and has 3D magnetic, thermal, electric, piezoelectric, and structural field capability with limited coupling between the fields. The electric loading can be applied on the SOLID5 element. The boundary conditions for the simply supported composite plate are displacement and bending moment equal to zero at the four edges of the plate. The convergence test for the finite element mesh was conducted by varying number of elements in the thickness from 4 elements to 12 elements. A typical finite element mesh is shown in Figure 2. The mode (1,1) natural frequency of the composite laminate plate varying with number of elements in the thickness is plotted in Figure 3. It was found that the convergence for the finite element analysis can be achieved with 12 elements along the thickness direction. The natural frequencies of the simply supported composite laminate plate were determined using Equation (16) and compared with the numerical results from the finite element method via ANSYS as shown in Table 1. It can be observed that the natural frequencies determined by the present approach (16) agree well with the finite element results with difference less than 1%.

### 4.1. Effect of Excitation Frequency

**Figure 4:**
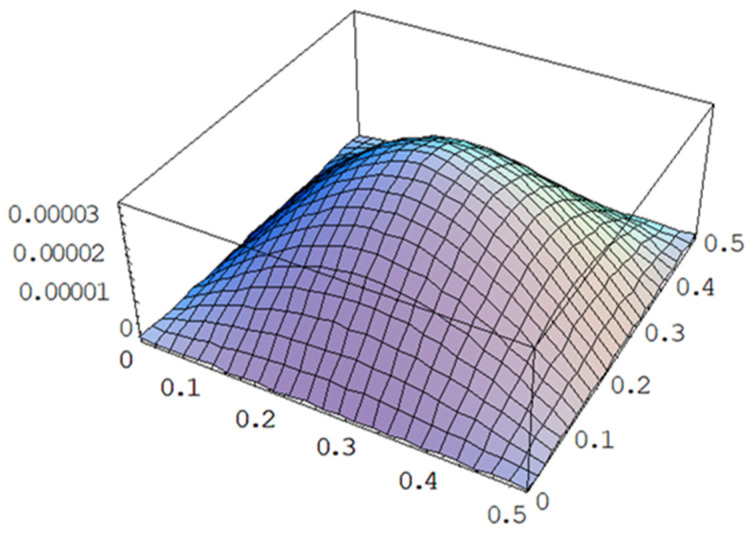
Vibrational amplitude (unit: mm) of a composite laminate plate excited by a PZT actuator with frequency of 143 Hz.

**Figure 5:**
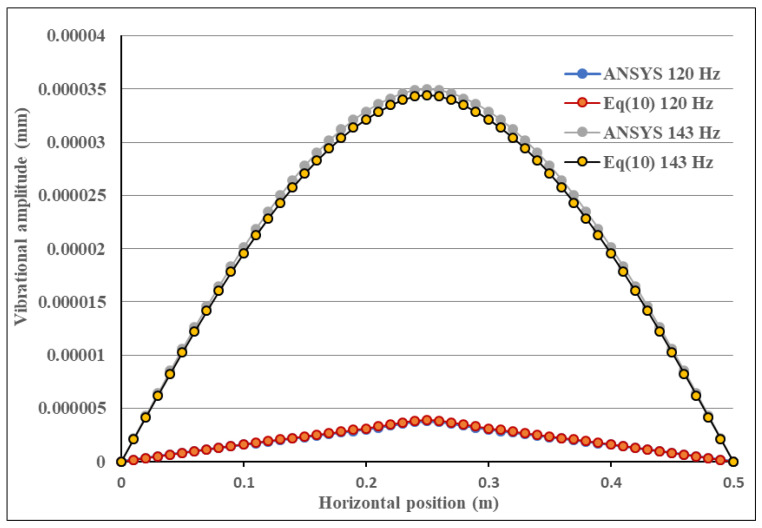
Vibrational amplitude distribution along the horizontal line (*y* = 0.25 m) of the composite laminate plate for the excitation frequencies of 143 Hz and 120 Hz.

**Figure 6:**
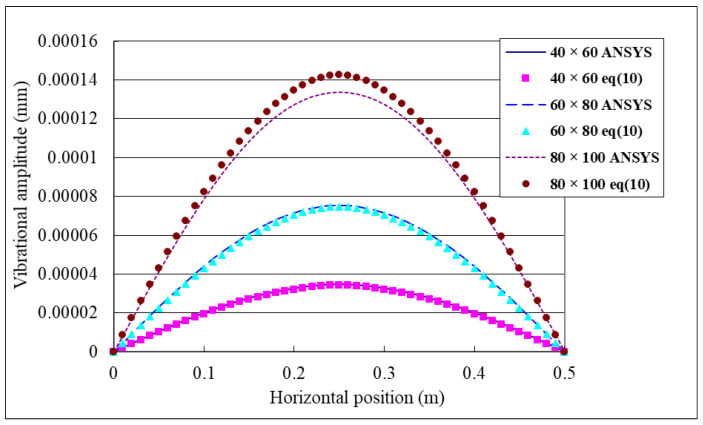
Vibrational amplitude distribution along the horizontal line (*y* = 0.25 m) of the composite laminate plate excited by PZT actuators with three different sizes.

PZT actuator with dimensions of 40 mm × 60 mm × 0.5 mm was attached on the center of the composite laminate plate. Two different frequencies 143 Hz and 120 Hz of the input voltage with amplitude of 100 V were applied on the PZT actuator. The frequency of 143 Hz is close to the mode (1,1) natural frequency 144.4 Hz of the composite laminate plate as shown in Table 1, while 120 Hz is off the natural frequency. The theoretical predictions of the vibrational responses of the composite laminate plate using Equation (10) were compared with the finite element results. Figure 4 illustrates the vibrational shape of the composite laminate plate excited by a PZT actuator at the frequency of 143 Hz. Vibrational amplitude distribution along the horizontal line (*y* = 0.25 m) of the composite laminate plate for the excitation frequencies of 143 Hz and 120 Hz are plotted in Figure 5. It can be seen that the vibrational amplitude for the excitation frequency of 143 Hz is much larger than that of 120 Hz. Thus, the mode (1,1) vibration of the composite laminate plate can be excited by a PZT actuator with frequency close to the natural frequency. The vibrational response predicted by the present approach Equation (10) is in good agreement with the finite element result. PZT actuators with different sizes were attached to the composite laminate plate to investigate the influence of PZT size on the vibrational response. Figure 6 shows the vibrational amplitude of the composite laminate plate excited by the PZT actuator with three different sizes, namely 40 mm × 60 mm × 0.5 mm, 60 mm × 80 mm × 0.5 mm and 80 mm × 100 mm × 0.5 mm, respectively. It appears that the vibrational amplitude increases as the PZT actuator size increases. The difference between the finite element result and the theoretical prediction is increasing as PZT size increases with a maximum difference of 6.7%. This may be attributed to the mass effect of the PZT actuator since the mass of the PZT actuator was neglected in the theoretical model.

### 4.2. Effect of PZT Location

**Figure 7:**
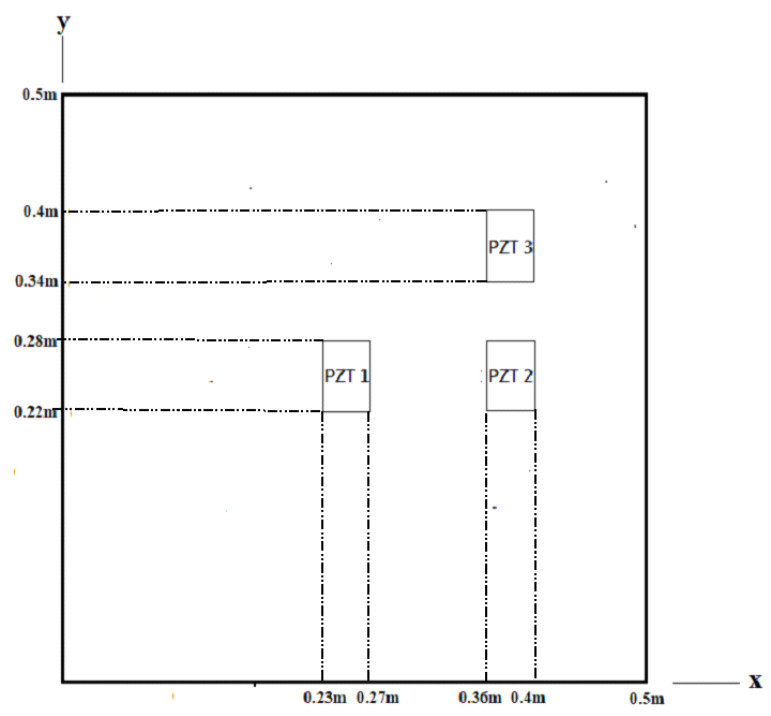
Three different locations of the surface-bonded PZT actuators.

**Figure 8:**
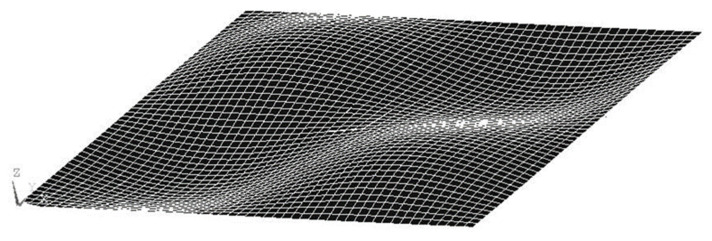
Mode (2,2) shape of a simply supported composite laminate plate.

**Figure 9:**
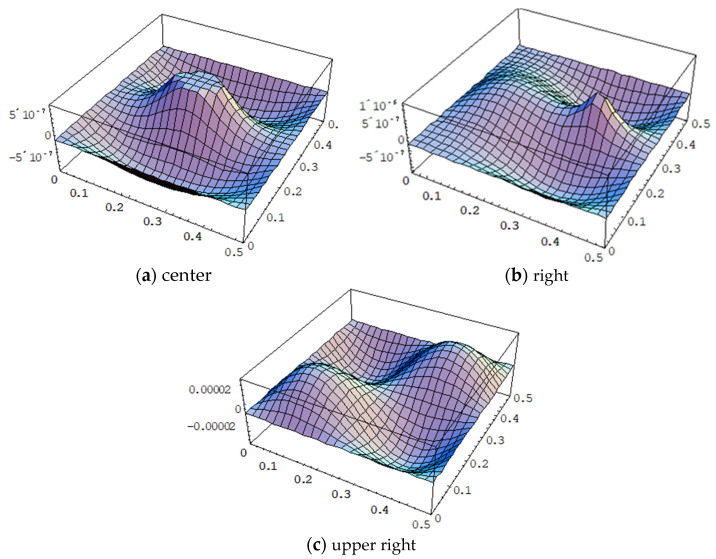
Vibrational amplitude (unit: mm) of the composite laminate plate excited by a PZT actuator bonded to three different locations.

It is essential to place the PZT actuator in an appropriate location to excite the desired vibration mode of a composite laminate plate. Three different locations of the PZT actuator attached on the center (PZT 1), right (PZT 2), and upper right (PZT 3) of the composite laminate plate as shown in Figure 7 were proposed to investigate the effect of PZT location on the vibrational response. A sinusoidal voltage with amplitude 100 V and frequency 578 Hz was employed on these PZT actuators separately to excite the mode (2,2) with the mode shape as shown in Figure 8. The vibrational shapes of the composite laminate plate excited by PZT 1, PZT 2, and PZT 3 actuators are presented in Figure 9a–c, respectively. It appears that the vibrational shape is heavily dependent on the location of the PZT actuator. The vibrational shape Figure 9c excited by the PZT 3 actuator located at the upper right is close to the mode (2,2) shape as shown in Figure 8. The PZT 3 actuator attached on the upper right of the composite laminate plate is located at the maximum amplitude of the mode (2,2) as shown in Figure 8. Thus, the mode (2,2) can be adequately excited by the PZT 3 actuator. The maximum vibrational amplitude $3.38\times 10^{-5}\;\mathrm{mm}$, excited by the PZT actuator attached on the upper right, is significantly larger than that of PZT actuators attached on the center and right, with maximum vibrational amplitudes of $1.71\times 10^{-6}\;\mathrm{mm}\;\mathrm{and}\;1.14\times 10^{-6}\;\mathrm{mm},$ respectively. This demonstrates that an appropriate location for the PZT actuator is required to successfully excite a certain mode.

### 4.3. Vibration Suppression

**Figure 10:**
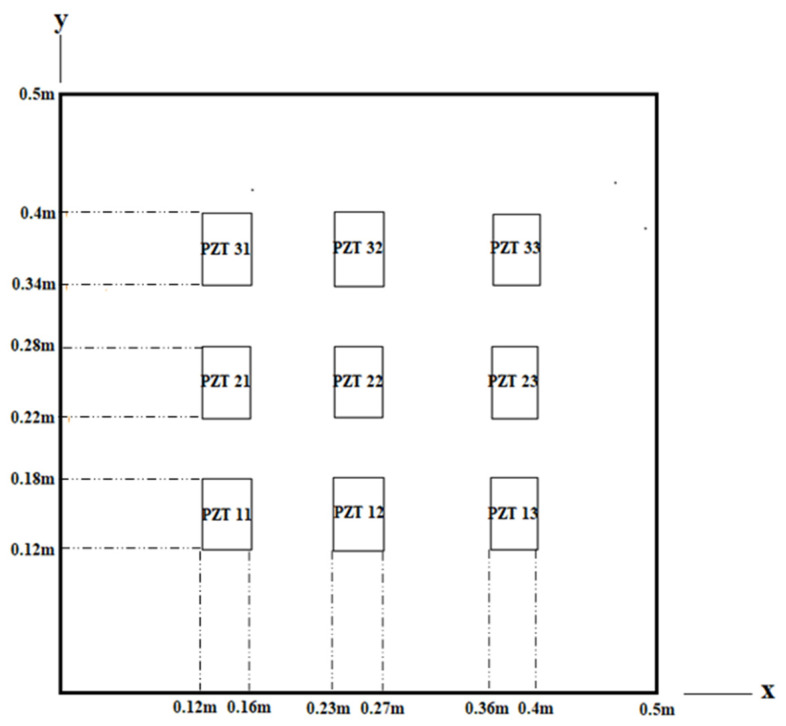
Periodic PZT 3 × 3 array on the composite laminate plate.

**Figure 11:**
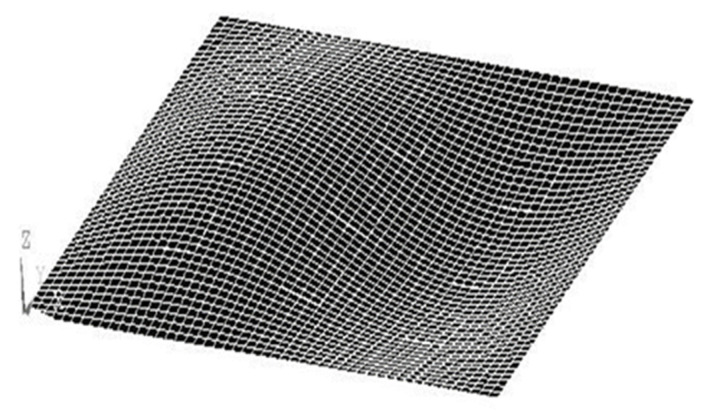
Mode (2,1) shape of the simply supported composite laminate plate.

**Figure 12:**
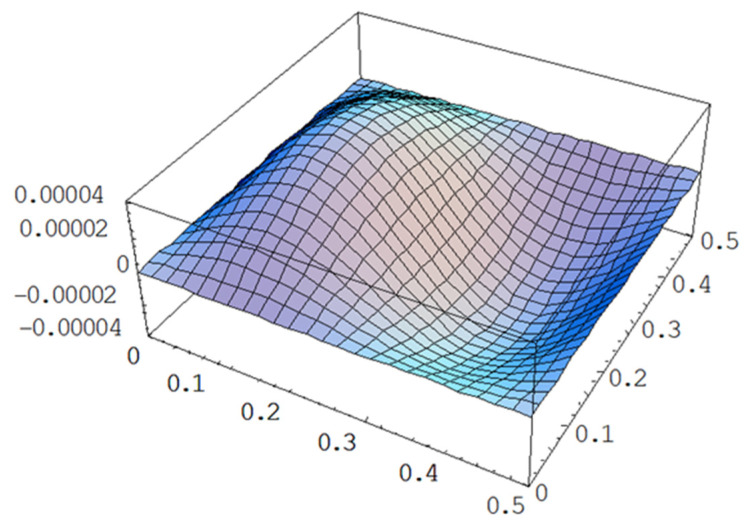
Vibrational amplitude (unit: mm) of a composite laminate plate excited by the PZT 21 actuator with frequency of 396 Hz.

**Figure 13:**
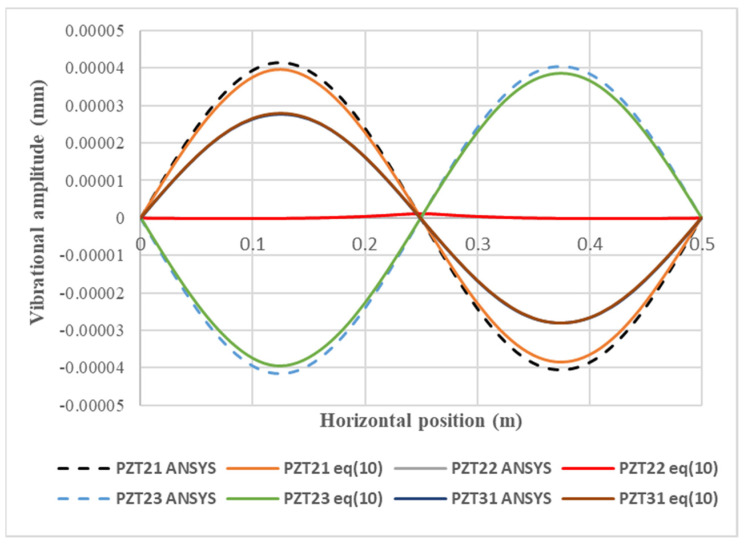
Vibrational amplitudes along the horizontal line (*y* = 0.25 m) of the composite laminate plate excited by the PZT 21, PZT 22, PZT 23, and PZT 31 actuators.

**Figure 14:**
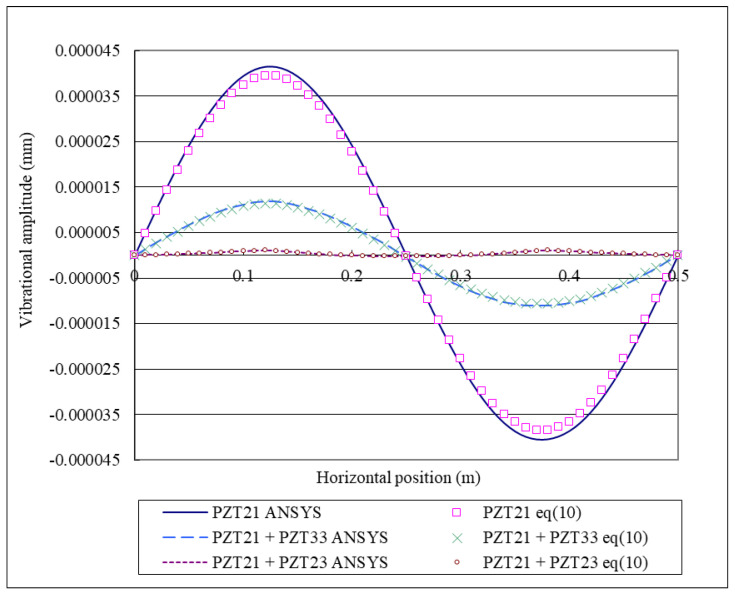
Vibrational amplitudes along the horizontal line (*y* = 0.25 m) of the composite laminate plate excited by the PZT 21, PZT 21 + PZT 23, and PZT 21 + PZT 33 actuators.

A PZT actuator not only can excite the vibration but also can be used to suppress the vibration. Multiple PZT actuators distributed in a 3 × 3 array as shown in Figure 10 were employed to suppress the vibration of the composite laminate plate. The natural frequencies of the composite laminate plate are listed in Table 1. The mode (2,1) shape with a natural frequency of 397.9 Hz is shown in Figure 11. A sinusoidal voltage with an amplitude of 100 V and frequency of 396 Hz was applied on the PZT 21 actuator. The vibrational amplitude of the composite laminate plate determined by Equation (10) is plotted in Figure 12, which is similar to the mode (2,1) shape as shown in Figure 10. The vibrational amplitudes along the horizontal line (*y* = 0.25 m) of the composite laminate plate excited by PZT 21, PZT 22, PZT 23, and PZT 31 actuators are presented in Figure 13. It can be seen that the vibrational amplitude of the composite laminate plate induced by the PZT 21 actuator is significantly higher than that of the PZT 22 and PZT 31 actuators. The vibrational amplitude excited by the PZT 22 actuator is approximately zero since PZT 22 is located at the nodal line of mode (2,1). These indicate that the mode (2,1) vibration can be excited by the PZT 21 actuator while the PZT 22 actuator is not able to excite the mode (2,1) vibration. The vibrational shape excited by PZT 23 is opposite to PZT 21. Thus, the mode (2,1) vibration excited by PZT 21 can be suppressed by introducing an opposite vibration from the PZT 23 actuator. In this work, the mode (2,1) vibration of the composite laminate plate was excited by PZT 12 actuator, and then PZT 23 actuator and the PZT 33 actuator were selectively activated to suppress the mode (2,1) vibration. Figure 14 plots the vibrational amplitudes along the horizontal line (*y* = 0.25 m) of the composite laminate plate induced by the PZT 21 actuator, the PZT 21 and PZT 23 actuators, and the PZT 21 and PZT 33 actuators. It can be seen that the vibrational amplitude is approximately zero as the PZT 23 actuator is activated to suppress the vibration that is excited by the PZT 21 actuator. The PZT 23 actuator can more effectively suppress the mode (2,1) vibration in comparison with the PZT 33 actuator. Moreover, the theoretical prediction Equation (10) is in good agreement with finite element results achieved using ANSYS software.

## 5. Conclusions

A theoretical model for the vibration excitation and suppression of a composite laminate plate by distributed PZT actuators attached on the structure was developed using composite mechanics and plate theory. The theoretical model has been employed to predict the vibrational response of a simply supported composite laminate plate excited by PZT actuators. The analytical solution was compared with the finite element method and found to be in good agreement. Numerical results show that as the input frequency becomes close to the natural frequency of a certain mode, the mode can be successfully excited provided the PZT actuator is placed at a proper position. The vibration shape is mainly dependent on the location and size of the PZT actuator when the input frequency is off the natural frequency. In addition, multiple PZT actuators arranged in an array with three rows and three columns were deployed on the composite laminate plate to excite a specific mode as well as suppress a certain mode. It was found that the efficiency of the PZT actuator to excite or suppress a specific mode is significantly affected by the location and frequency. The novelty of this work is that the complicated coupling problem between the composite plate and attached PZT actuator is resolved into two simple problems. First, loads (bending moment) induced by the PZT actuator to the composite plate are derived. Then, a simple analytical solution for the vibrational response of a composite plate subjected to the bending moment is developed. The novel methodology of the proposed model, which theoretically predicts the vibrational response of a composite plate excited by PZT actuators, can be used to study a variety of applications of PZT actuators such as surface cleaner, piezoelectric motor, energy harvester, and noise attenuation.

## Figures and Tables

**Table 1:** Natural frequencies (Hz) of a simply supported composite laminate plate.

Mode (*m*,*n*)	ANSYS	Equation (16)	Difference
(1,1)	144.4	145.0	0.42%
(1,2)	340.4	340.2	0.06%
(2,1)	397.9	397.6	0.08%
(2,2)	580.3	579.9	0.07%
(3,1)	673.2	672.3	0.13%

## Data Availability

Data are available on request.
